# Functional verification of computationally predicted qnr genes

**DOI:** 10.1186/1476-0711-12-34

**Published:** 2013-11-21

**Authors:** Carl-Fredrik Flach, Fredrik Boulund, Erik Kristiansson, DG Joakim Larsson

**Affiliations:** 1 Department of Infectious Diseases, University of Gothenburg, Gothenburg, Sweden; 2 Department of Mathematical Statistics, Chalmers University of Technology, Gothenburg, Sweden

**Keywords:** *qnr*, Vfu*qnr*, *assembled qnr 1*, Functional verification, Recombinant expression, *E. coli*

## Abstract

**Background:**

The quinolone resistance (*qnr)* genes are widely distributed among bacteria. We recently developed and applied probabilistic models to identify tentative novel *qnr* genes in large public collections of DNA sequence data including fragmented metagenomes.

**Findings:**

By using inducible recombinant expressions systems the functionality of four identified *qnr* candidates were evaluated in *Escherichia coli*. Expression of several known *qnr* genes as well as two novel candidates provided fluoroquinolone resistance that increased with elevated inducer concentrations. The two novel, functionally verified *qnr* genes are termed Vfu*qnr* and *assembled qnr 1*. Co-expression of two *qnr* genes suggested non-synergistic action.

**Conclusion:**

The combination of a computational model and recombinant expression systems provides opportunities to explore and identify novel antibiotic resistance genes in both genomic and metagenomic datasets.

## Findings

Expression of *qnr* genes protects type II topoisomerases from quinolone inhibition, and facilitates the selection of mutants with higher level of resistance [1,2]. Six families of plasmid-borne *qnr* genes have been identified; *qnrA*, *B*, *C*, *D*, *S* and *VC*[1,3-7]. In addition, chromosomal *qnr* alleles have been found in several bacterial species, alleles that may serve as progenitors of plasmid-borne *qnr* genes [8-10].

Identifications of the first representative of each *qnr* family relied on screening of clinical isolates and resistance transfer assays [1,3-5,7,11], whereas subsequent studies have identified related *qnr* genes mainly by using PCR-based strategies [5,9,10,12]. Lately, several *qnr* genes have been discovered based on sequence homology by use of alignment tools [11,13]. However, this approach does not efficiently take into account the conserved pentapeptide repeat pattern of *qnr* genes, neither is it suitable for identifying *qnr* genes in short-read metagenomic datasets. The lack of optimized methods suggests that there very well could be many *qnr* genes waiting to be discovered. We have recently developed a computational method based on probabilistic Hidden Markov models with a potential to overcome these hurdles. By using this method a number of putative *qnr* genes were identified from a large collection of genomes and metagenomes [14].

In this study we have experimentally evaluated four of these novel candidates (NC), *nc1* – *nc4* (Additional file 1), in recombinant *Escherichia coli* expression systems, an approach that has been used with success to characterize *qnr* genes previously [3-5,7,8,13,15]. The candidates and the first members of the six described families of mobile *qnr* genes were synthesized (Eurofins MWG Operon, Ebersberg, Germany) and subcloned into two expression vectors, pZE21 and pZA14 (Expressys, Ruelzheim, Germany), under the control of two different inducible promoters, P_LtetO-1_ and P_lac/ara-1_ respectively. The vectors carry either the ColE1 (pZE21) or the p15A (pZA14) origin of replication, and thus belong to different plasmid compatibility groups [16]. Although several cloning procedures were tested, both by us and a contract lab, *nc4* could not be subcloned into the pZA14 vector and was thus not evaluated under the control of the P_lac/ara-1_ promoter. However, this was not likely due to toxicity of the gene product, as has been reported for EfsQnr previously [17], since *nc4* could be evaluated in the *E. coli* host using the pZE21 vector. The recombinant plasmids were electroporated into *E. coli* C600Z1 (Expressys), which expresses repressors for the two inducible promoters used [16]. Conceivably, these kinds of expression systems allow the evaluation of gene candidates without knowledge of the intrinsic promoter, information that is often difficult to capture when genes are reconstructed from fragmented metagenomes. In addition, the approach does not depend on the functionality of an intrinsic promoter in a heterologous expression host.

**Figure 1:**
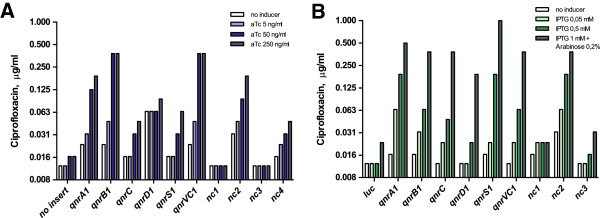
**Ciprofloxacin MICs for recombinant *E. coli *strains carrying known *qnr *genes or novel candidates (*nc1-4*).** The investigated genes are situated on pZE21 **(A)** or pZA14 **(B)**, and expression was stimulated by adding indicated concentrations of the inducers aTc **(A)** or IPTG/Arabinose **(B)**.

In order to investigate the genes’ capacity to confer fluoroquinolone resistance to the *E. coli* host, different concentrations of expression inducers were added to the Mueller-Hinton growth media used for fluoroquinolone susceptibility determinations with Etest® gradient strips (BioMérieux SA, Marcy l’Etoile, France). When the genes were evaluated under the control of the anhydrotetracycline (aTc)-inducible promoter P_LtetO-1_, all strains carrying known *qnr* genes showed ciprofloxacin minimal inhibitory concentrations (MICs) that increased with elevated inducer concentration (Figure 1A). In addition, strains carrying the novel *qnr* candidates *nc2* and *nc4* showed similar changes in ciprofloxacin susceptibility. All genes providing increased ciprofloxacin MICs after addition of aTc also conferred resistance when evaluated in the recombinant expression system controlled by the IPTG and arabinose-induced P_lac/ara-1_ promoter (Figure 1B). In the latter system a marginal MIC increase was observed also for *nc1* and *nc3* after addition of inducer, however a similar small increase was also observed for the control carrying the non-related luciferase gene instead of any *qnr* gene. Noteworthy, the choice of inducible promoter determined which *qnr* genes that gave rise to the strongest resistance phenotype, confirming the importance of genetic context. Comparable results were obtained when moxifloxacin susceptibility was evaluated after induced expression of the four candidates, *nc2* and *nc4* being the two that conferred fluoroquinolone resistance to the host (Additional file 2).

**Figure 2:**
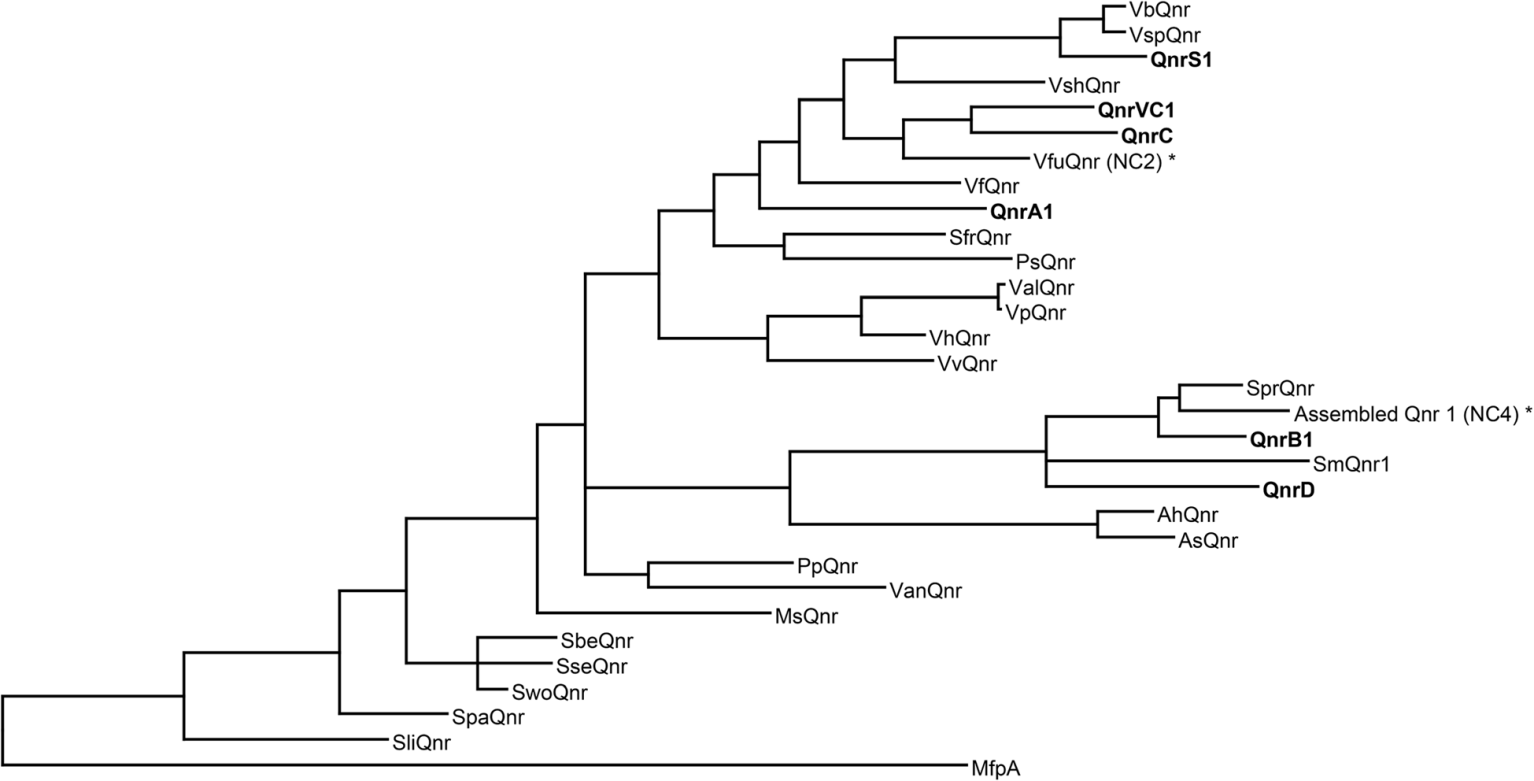
**Phylogenetic tree showing the relationships between Qnr variants.** The six plasmid-borne classes are shown in bold. The two candidates functionally verified in this work are marked with an asterisk (NC2 and NC4). The tree was constructed using MrBayes [23].

Although recombinant expression in *E. coli* could not verify *nc1* and *nc3* as functional *qnr* genes, it does not rule out functionality in another host. The *nc1* is relatively dissimilar to all known *qnr* genes (33% amino acid identity to QnrC and QnrS1) and originates from a coastal sea water metagenome, suggesting that the natural bacterial host might be distantly related to *E. coli*. The *nc2* gene was identified in the sequenced genome of *Vibrio furnissii*[18] and its deduced protein product shows 72% identity to QnrC and QnrVC1 (Figure 2). When the *nc4* gene was identified in baby stool metagenomes, its gene product showed the highest similarity to QnrB determinants identified in *Citrobacter freundii* (up to 80% identity) [9]. Later, sequences from *Serratia marcescens* encoding a protein, not verified to confer resistance, showing 98% identity with NC4 have been submitted to GenBank [19]. Only three amino acids between position 142 and 146 differ between NC3 and NC4. Our results indicate that this region is of importance for the protein’s functionality, at least in *E.coli*. The two candidates show 78-79% identity to QnrB1 (Figure 2), for which two loop structures are important for the quinolone inhibiting action [20]. In addition, mutational analyses of QnrB1 have identified several individual amino acids critical for its protective activity [21,22]. However, amino acids 142–146 are not located within the loop structures and were not included or identified as critical in the mutational analyses.

**Figure 3:**
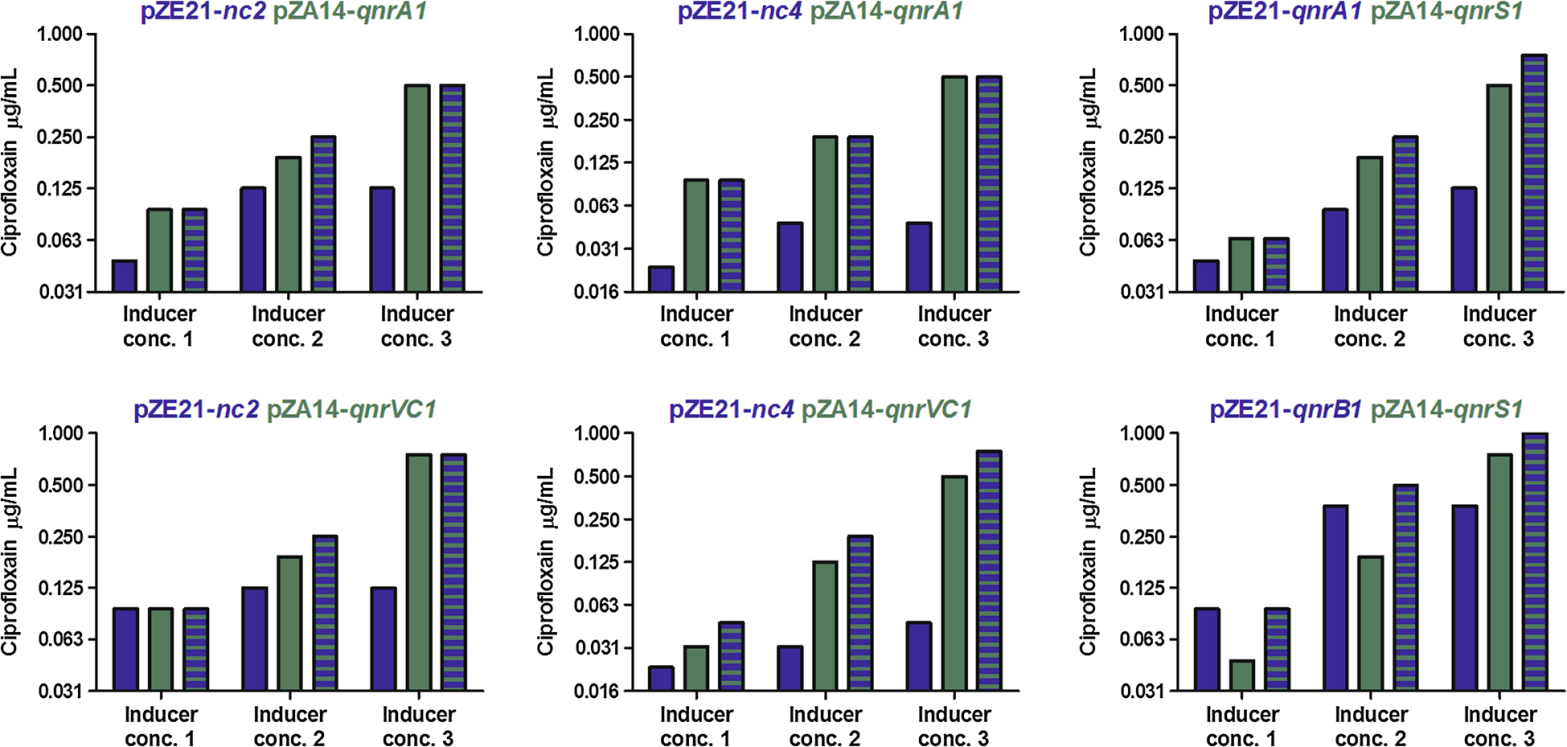
**Ciprofloxacin susceptibility of recombinant *E. coli *strains carrying two different *qnr *genes.** MICs of ciprofloxacin were determined after addition of inducers at three different concentrations described in Figure 1, which stimulated expression from the pZE21 plasmid (blue bars), the pZA14 plasmid (green bars), or both (striped bars). The header of each graph describes the *qnr* content of each strain and from which plasmids they are expressed.

Various bacteria, including *E. coli*, may carry two different *qnr* genes [24,25]. The benefit of this is not known. We therefore constructed recombinant strains containing two *qnr* genes situated on different plasmids (pZE21 and pZA14). We evaluated six different combinations of *qnr* genes including *qnrS* together with either *qnrA* or *qnrB*, which are two of the combinations observed in clinical isolates [24,25]. After induction with three different concentrations of inducers, at the most, a marginal increase in ciprofloxacin MIC (less than two-fold) was observed when both *qnr* genes were induced (Figure 3). This suggests a non-synergistic effect, which is in line with the assumed mode of action of Qnr proteins as well as with earlier susceptibility determinations showing that isolates carrying two *qnr* genes do not display a superior resistance phenotype [24,25].

We suggest that the two identified and experimentally verified *qnr* genes *nc2* and *nc4*, which provide resistance to the *E.coli* host at the same level as previously reported *qnr* genes, should be termed Vfu*qnr* and *assembled qnr 1*, respectively (Figure 2). The name Vfu*qnr* reflects the host of the gene, whereas the latter name is chosen to reflect that the gene is assembled from metagenomic DNA, and that the host is not known. The nucleotide sequences of Vfu*qnr* and *assembled qnr 1* have been submitted to GenBank (accession number BK008765 and KF278752, respectively).

To conclude, we have identified two novel, functional quinolone resistance genes. The development of a probabilistic Hidden Markov model and inducible recombinant expression systems together provided an efficient approach for exploring bacterial genomes and fragmented metagenomic datasets for the presence of novel *qnr* genes. We propose that a similar concept could be developed and used to identify resistance genes for other classes of antibiotics as well.

## Availability of supporting data

The data sets supporting the results of this article are included within the article and its additional files.

## Competing interests

The authors declare that they have no competing interests.

## Authors’ contributions

CFF designed and performed the cloning and antibiotic susceptibility determination experiments as well as drafted the manuscript. FB performed the bioinformatic analyses and revised the manuscript. DGJL and EK conceived the study and revised the manuscript. All authors read and approved the final manuscript.

## Supplementary Material

Additional file 1: Table S1 Nucleotide sequences of the *novel candidate* (nc) genes. Click here for file

Additional file 2: Figure S1 Moxifloxacin MICs for recombinant *E. coli* strains carrying known *qnr* genes or novel candidates (nc1‒4). The investigated genes are situated on pZE21 (A) or pZA14 (B), and expression was stimulated by adding indicated concentrations of the inducers aTc (A) or IPTG/Arabinose (B). Click here for file
