# Supplementary material for: Functional verification of computationally predicted qnr genes
Source: Ann Clin Microbiol Antimicrob. 2013 Nov 21;12:34. doi: 10.1186/1476-0711-12-34 (PMC4222258; doi:10.1186/1476-0711-12-34)
Supplement: Additional file 1: Table S1 — Nucleotide sequences of the novel candidate (nc) genes. [file 1476-0711-12-34-S1.pdf]

TABLE S1. Nucleotide sequences of the *novel candidate* (*nc*) genes

nc1	originates from metagenome (MG-RAST: 4441580)
1	ATGGGTGAGGTAATGGACATACAAATAGGCGAAGGCTATACCAACTTAGATCTGCGCAAT
61	AAGGACTTCAGCGGCCAAGATTTAACCGACTGCACCTTTAGCCAATGCCAATTTGCGGGC
121	AGTGTCTTCCGCGGCGCCAACCTGACTAACTGTCAGTTTGATCGATGCCAATTCAACAAC
181	AGTGATCCAGACGAGCCCGCAAGCTTTGAATGGGCAAACTTGCGCGAAGCGGTGTTTAGC
241	CATTGCGACCTAAACATGGTGGCCTTCAATCATTGCCTGGGTTACGACCTGAGCTTTAAA
301	GAATGTCAAATGCAGGGCGCAGATTTATCTCTAGGAGATTTCAAAATGCCTGTAGGCAAT
361	CTGGATTTGGCCGCACTGACCATTACCGACTGTAATTTTTCCTACGGCAATTTATCCAAT
421	ACCTTTTTGGTTGGCTGCAAGCTTTCCAACAATCGACTAACTGAATGCTTGTTAGATTTT
481	GCAGATCTAAGTGATGCGGATTTAAGCGACAGCGAAATGTTTAATGTTTCAGCACGCAAC
541	TGCAAACTCAAAGGCGCAGACTTGCGCGGTGCAAGCTTCAACAACTTAGATTTGAAAAGC
601	TTAGACCTAGAGGGTGTGCGCCTGTACTACAACCAGTTACCTGCGTTAGTAGATTGA
nc2	originates from *Vibrio furnissii* genome (CP002378.1)
1	ATGAAATTAAACCATCACACTTACCAACACACCGATTTCTCAAATCGGGATTTGTCGAAC
61	TTAGTTTTCAACGATTGTTGCTTCTATCAATGCAACTTTAGCCGTGCCGACATGCGTGAT
121	TCCAAATTCATCAATTGCAGCTTTATTGAGAAAAGCGATGTGGAGGGATGTAATTTTGCG
181	TATGCCGATCTCAGAGATGCGTCCTTTCAACACTGCCGATTGGCGATGGCTCATTTCAGT
241	GGGGCGGACTGTTTTGGAATTGAACTGAGAGATTGTGATTTAAAAGGTGCCAATTTTTCT
301	CGCGCCCGCTTTGTTAATCAGGTCTCGAACAAAATGTTTTTTTGTTCCGCGTACATCACG
361	GGATGTAATTTATCCTATGCCAACTTGGAGCGGCAGTGCCTCGAAAAATGTGATCTGTTT
421	GAAAATCGCTGGATTGGCGCCAACTTGCACGGGGTTTCTTTTAAAGAGTCTGATTTAAGC
481	CGGGGTTCATTTTCAGAGGACTGCTGGGAACAAGCGCGCTTCCAAGGTTGTGACTTAACC
541	CATATTGAACTGACCGGGTTGGATCCCCGCAGAGTGGATTTAACGGGGGTAAAAATTTGC
601	GATTGGCAGCAAGAACAACTGCTAGAGAAGCTGGGTGTGATTGTGGTTCCGGACTAA
nc3	originates from metagenome (SRA: SRX032366)
1	ATGAGTCTGATGTTGAAGGGTGAAAAGATTGACCGTAACCGCTTTACCGGCGAGAAGATT
61	GAAAACGGCAGCTTTATGCTGTGTGATTTTTCGGGCGCCGATCTGACCGGCACCGAGTTT
121	ATCGGCTGCCAGTTTTACGATCGTGACAGCCGACAGGGCGGCAATTTCAGCCGTGCGATA
181	CTCAAAGACGCCAGCTTCAGAAGCTGCGATTTGTCGATGGCCGACTTCCGCTACGCCAGC
241	GCCCTGGGGCTGGAGATCCGCGAATGCCGCGCGCAGGGCGCCGATTTTCGCGGCGCCAGT
301	TTTATGAACATGATCACCAGCCGCATCGGGTTTTGCAGCGCCTATATCACCAAAAGCAAC
361	CTGAGCTACGCCAACTTCGCCAAGGTGGTGCTGGAGAAATGCGAGCTGTGGGAAAACCGC
421	TGGCAAGGCACACAGGGACTCGGCGCCAGCTTCAGCGGCTCCGATCTGTCCGGCGGCGAG
481	TTCTCCGGATTCGACTGGCGCGCCGCCGACGTCACCCAGTGCGATTTGAGCAATGCGGAA
541	CTGGGCGAATTGGATTTACGCACCACCGATCTGCAGGGCGTCAAAATGGACAGCCACCAG
601	GCCGCGCAGCTGCTGGAGCGGCTGGGGATCGCGATCGTCGGCTGA
nc4	originates from metagenome (SRA: SRX032366)
1	ATGAGTCTGATGTTGAAGGGTGAAAAGATTGACCGTAACCGCTTTACCGGCGAGAAGATT
61	GAAAACGGCAGCTTTATGCTGTGTGATTTTTCGGGCGCCGATCTGACCGGCACCGAGTTT
121	ATCGGCTGCCAGTTTTACGATCGTGACAGCCGACAGGGCGGCAATTTCAGCCGTGCGATA
181	CTCAAAGACGCCAGCTTCAGAAGCTGCGATTTGTCGATGGCCGACTTCCGCTACGCCAGC
241	GCCCTGGGGCTGGAGATCCGCGAATGCCGCGCGCAGGGCGCCGATTTTCGCGGCGCCAGT
301	TTTATGAACATGATCACCAGCCGCATCGGGTTTTGCAGCGCCTATATCACCAAAAGCAAC
361	CTGAGCTACGCCAACTTCGCCAAGGTGGTGCTGGAGAAATGCGAGCTGTGGGAAAACCGC
421	TGGCACGGCGCACAGGTGCTCGGCGCCAGCTTCAGCGGCTCCGATCTGTCCGGCGGCGAG
481	TTCTCCGGATTCGACTGGCGCGCCGCCGACGTCACCCAGTGCGATTTGAGCAATGCGGAA
541	CTGGGCGAATTGGATTTACGCACCACCGATCTGCAGGGCGTCAAAATGGACAGCCACCAG
601	GCCGCGCAGCTGCTGGAGCGGCTGGGGATCGCGATCGTCGGCTGA
