# Supplementary material for: Functional verification of computationally predicted qnr genes
Source: Ann Clin Microbiol Antimicrob. 2013 Nov 21;12:34. doi: 10.1186/1476-0711-12-34 (PMC4222258; doi:10.1186/1476-0711-12-34)
Supplement: Additional file 2: Figure S1 — Moxifloxacin MICs for recombinant E. coli strains carrying known qnr genes or novel candidates (nc1‒4). The investigated genes are situated on pZE21 (A) or pZA14 (B), and expression was stimulated by adding indicated concentrations of the inducers aTc (A) or IPTG/Arabinose (B). [file 1476-0711-12-34-S2.pdf]

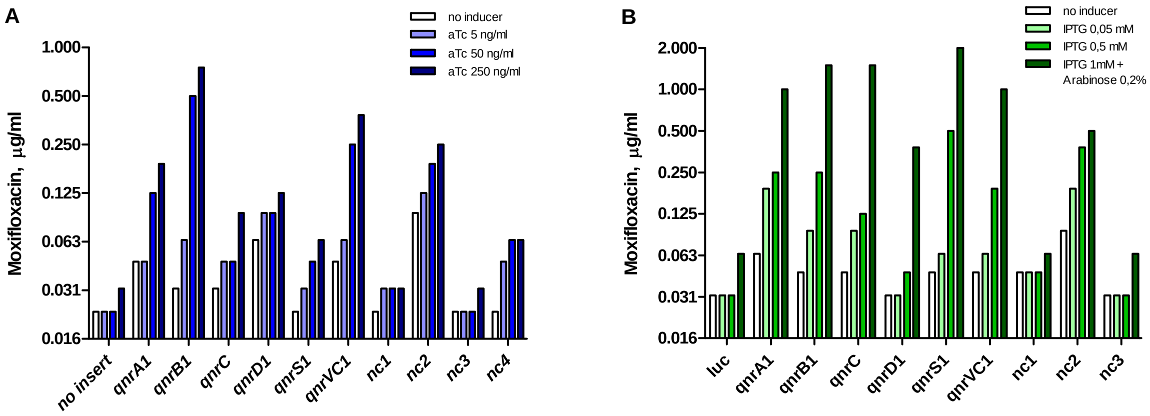

Figure S1. **Moxifloxacin MICs for recombinant *E. coli* strains carrying known *qnr* genes or novel candidates (*nc1-4*).** The investigated genes are situated on pZE21 (A) or pZA14 (B), and expression was stimulated by adding indicated concentrations of the inducers aTc (A) or IPTG/Arabinose (B).
